# Kounis syndrome caused by bee sting: a case report and literature review

**DOI:** 10.5830/CVJA-2022-042

**Published:** 2022-08-29

**Authors:** Wen-Juan Lin, Yue-qing Zhang, Zhen Fei, Dan-dan Liu, Xing-Hang Zhou

**Affiliations:** Department of Cardiology, The First Affiliated Hospital of Zhejiang University, Hangzhou, China; Department of Cardiology, Changxing Hospital of Traditional Chinese Medicine, Huzhou, China; Department of Internal Medicine, Hengdian Hospital, Dongyang, China

**Keywords:** Kounis syndrome, China, bee sting

## Abstract

Kounis syndrome is defined as an acute coronary syndrome (ACS) secondary to allergic or hypersensitivity reactions. It can be further categorised into subtypes such as coronary vasospasms, acute myocardial infarction or stent thrombosis based on the pathogenesis. Kounis syndrome is most likely an underdiagnosed condition in China, given the many triggers reported in the literature. Herein, we report a case of Kounis syndrome, possibly triggered by a bee sting. The patient had late onset of angina symptoms with delayed diagnosis due to unfamiliarity with this condition. In patients with clinical signs of ACS that are superimposed on a hypersensitivity reaction, especially those with pre-existing cardiovascular risk factors, Kounis syndrome should be considered, so that appropriate assessment and treatment can be initiated. Prompt management of both the allergic reaction and the ACS is vital for Kounis syndrome.

It is well known that cardiovascular symptoms can arise after allergic, hypersensitivity, anaphylactic or anaphylactoid reactions. The first report of acute myocardial infarction after urticaria associated with penicillin use was published in 1950.1 However, it was not until 1991 that the term allergic angina syndrome or Kounis syndrome was proposed to describe the occurrence of an acute coronary syndrome (ACS), that is, angina and myocardial infarction, as a result of endothelial dysfunction secondary to allergic reaction. These ACS events may occur in the form of coronary spasms, acute myocardial infarction or stent thrombosis.^2^

Kounis syndrome has been reported in different ethnic and age groups (from two- to 90-year-olds) and geographic locations. Available data suggest that Kounis syndrome might not be a rare disease but is under reported in the literature due to being missed or undiagnosed in clinical practice. As far as China is concerned, there are only a few case reports so far concerning this entity. Besides, in the majority of cases, the time between trigger event and symptom onset is usually within one hour, which is not surprising given the nature of the acute immune response.

Herein, we report a case of Kounis syndrome, possibly triggered by a bee sting. However, the patient had late onset of angina symptoms with delayed diagnosis due to unfamiliarity with this condition. Therefore, this case should raise awareness of the existence of Kounis syndrome, especially for physicians practicing at primary care facilities in China.

## Case report

A 42-year-old male was referred to our emergency department (ED) with a three-day duration of progressively worsening retrosternal chest pain following upper limb joint pain, and a reddish swollen skin area behind the right ear that had occurred after a bee sting. His past medical history included poorly controlled hypertension for 10 years, being a smoker of more than 40 pack-years, and an allergy to insects on skin contact.

The initial bee sting happened in the morning and was behind the right ear, which caused local skin allergic reactions of tenderness, redness and swelling (1 × 1 cm) and he stated he had removed the stinger by himself (day one). The next morning, he developed bilateral shoulder and elbow joint pain, which was aggravated by movement. The swollen skin area also increased to 3 × 3.5 cm. Later the same day (34 hours after the sting), he started to have retrosternal cardiac type chest pain, which gradually worsened, with accompanied diaphoresis.

**Fig. 1:**
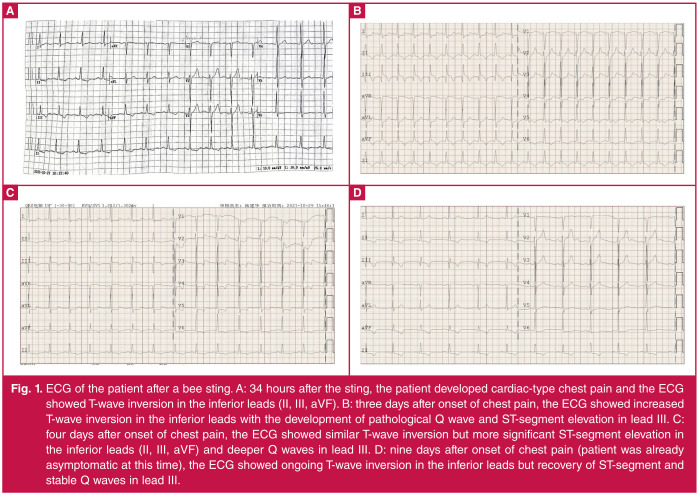
ECG of the patient after a bee sting. A: 34 hours after the sting, the patient developed cardiac-type chest pain and the ECG showed T-wave inversion in the inferior leads (II, III, aVF). B: three days after onset of chest pain, the ECG showed increased T-wave inversion in the inferior leads with the development of pathological Q wave and ST-segment elevation in lead III. C: four days after onset of chest pain, the ECG showed similar T-wave inversion but more significant ST-segment elevation in the inferior leads (II, III, aVF) and deeper Q waves in lead III. D: nine days after onset of chest pain (patient was already asymptomatic at this time), the ECG showed ongoing T-wave inversion in the inferior leads but recovery of ST-segment and stable Q waves in lead III.

**Fig. 2:**
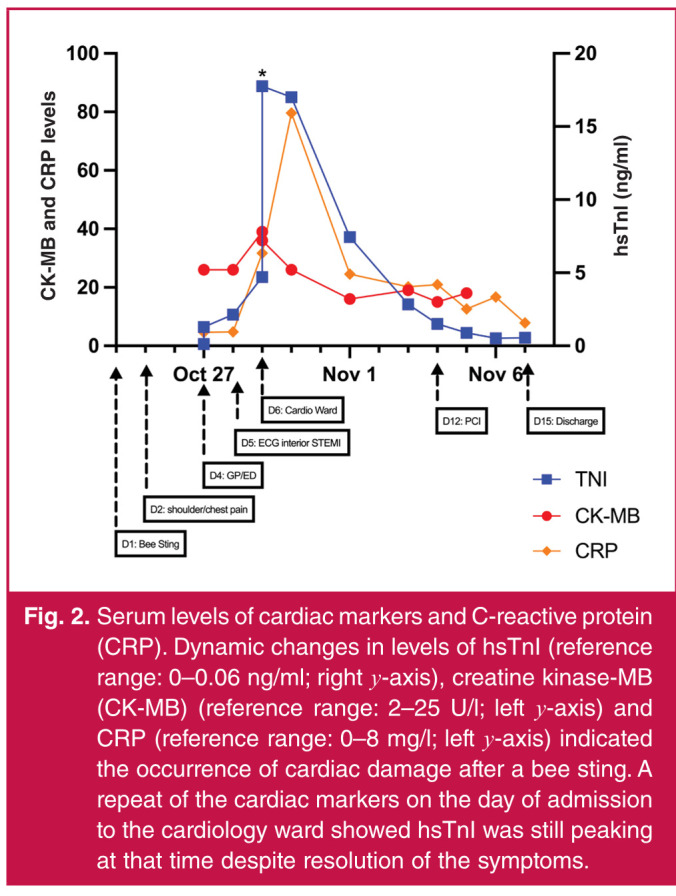
Serum levels of cardiac markers and C-reactive protein (CRP). Dynamic changes in levels of hsTnI (reference range: 0–0.06 ng/ml; right y-axis), creatine kinase-MB (CK-MB) (reference range: 2–25 U/l; left y-axis) and CRP (reference range: 0–8 mg/l; left y-axis) indicated the occurrence of cardiac damage after a bee sting. A repeat of the cardiac markers on the day of admission to the cardiology ward showed hsTnI was still peaking at that time despite resolution of the symptoms.

He consulted a local general practitioner on day four (about 36 hours after onset of chest pain) who recorded high blood pressure (260/160 mmHg) and T-wave inversion in the inferior leads on electrocardiogram (ECG) (Fig. 1A). He was prescribed metoprolol and nifedipine and urgently referred to a local hospital ED the same day, where laboratory investigations showed high glucose (27.30 mmol/l) and elevated high-sensitivity troponin I (hsTnI) levels (155.9 ng/l, reference range 0–40) (Fig. 2).

He was transported to our ED via road ambulance the same night. On arrival, his vitals were: heart rate 89 beats per minute, blood pressure 171/110 mmHg, respiratory rate 18 breaths per minute and temperature 37.3°C. Laboratory investigations showed rising hsTnI and elevated NT-proBNP levels (Fig. 2).

A transthoracic echocardiogram (TTE) showed left ventricular (LV) segmental dysfunction (inferior, posterior and lateral wall systolic dysfunction, estimated LV ejection fraction of 45%). A thoracic and abdominal computed tomography (CT) aortogram to rule out aortic dissection was unremarkable. Therefore, the provisional diagnosis at that time was allergic myocarditis due to bee sting toxin with a differential diagnosis of ACS. The patient was treated with a single antiplatelet agent (aspirin) for ACS, antihypertensives, an anti-inflammatory (methylprednisolone), and atorvastatin for plaque stabilisation. Serial ECG the next day (day five) suggested the development of interior ST-segment elevation myocardial infarction (increased T-wave inversion of II/III/aVF leads, ST elevation with pathological Q waves on lead III) (Fig. 1B, C).

He was then admitted into the cardiology ward on day six, by which time cardiac symptoms had largely resolved despite peaking hsTnI and NT-proBNP levels (Fig. 2). Antihistamine (loratadine) was added and basal-bolus insulin was given for the likely undiagnosed type 2 diabetes (haemoglobin A1c was 10.0%). Elevation of complement C3 was also detected (Fig. 2). A serial ECG monitor showed gradual recovery of ST elevation in the next few days with persistent pathological Q- and T-wave inversion (Fig. 1D).

**Fig. 3:**
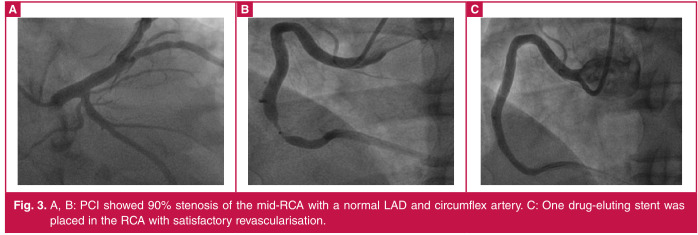
A, B: PCI showed 90% stenosis of the mid-RCA with a normal LAD and circumflex artery. C: One drug-eluting stent was placed in the RCA with satisfactory revascularisation.

Elective percutaneous coronary intervention (PCI) on day 12 showed 90% stenosis of the mid-right coronary artery (RCA) with a normal left anterior descending artery (LAD) and circumflex artery (Fig. 3). One drug-eluting stent was placed in the RCA and the patient had an uneventful recovery. Repeat TTE showed improved LV ejection fraction to 55%. The patient was discharged three days post PCI with a prescription of dual antiplatelet therapy (aspirin + clopidogrel), atorvastatin, metoprolol, nifedipine and basal-bolus insulin therapy.

## Discussion

Kounis syndrome is the occurrence of ACS after an allergic insult, the latter of which can be hypersensitivity, anaphylactic or anaphylactoid conditions. The underlying mechanism has been attributed to mast cell (histamine) and platelet (platelet-activating factor) activation and interaction with other inflammatory cells such as macrophages and T lymphocytes, with involvement of a range of cytokine cascades, such as activation of the complement system (C3a, C5a).3 The end effect is coronary artery spasm and/or atheromatous plaque erosion or rupture. To date, three variants have been proposed, coronary spasm, acute myocardial infarction and stent thrombosis.

Since its description, the list of allergic triggers for Kounis has been ever expanding. Medications, especially antibiotics, represent the most common iatrogenic trigger overall. In contrast, non-steroidal anti-inflammatory drugs were the most frequent trigger drugs for cases in the USA.^4^ Other trigger factors include various food and environmental exposures such as pollen and insect bites (bee sting).^5^

Given the ubiquitous presence of triggers, Kounis syndrome might not be a rare disease. However, there is a paucity of data on its prevalence and incidence, as the most relevant literature is case reports only. One study estimated the incidence of Kounis syndrome in the ED among all allergy patients in that study year to be 3.4%.^3^

When it comes to China, the country with the largest population, the number of case reports is surprisingly low. For Chinese patients, the triggers that have been reported include, but are not limited to, drug‑eluting stents,^6^ antibiotics,^7^ hormone pills,^8^ oral traditional Chinese medicines (TCM),^9^ intravenous cervus and cucumis polypeptides,^9^ intramuscular anisodamine,^10^ as well autoimmune conditions.^11^-^13^

This low number of cases reported in the Chinese population is in sharp contrast with the fact that most Kounis syndrome cases have been reported in southern Europe.^3^ This difference could well be attributed to increased awareness of physicians of the existence of Kounis syndrome in that geographic area, in addition to other factors such as differences in the prevalence of environmental triggers, medication prescription/consumption behaviour and genetic predisposition to an allergic response.

One unique aspect of medical practice in China is the prescription of herbal TCM, both in oral and intravenous form, especially in the vast rural and regional areas. TCM injections have been associated with various adverse drug reactions, including hypersensitivity and anaphylactic shock.^14^-^16^ Besides, there have also been inappropriate antibiotic prescriptions, especially in Chinese primary healthcare facilities.^17^ Sadly, there is a recent case report of Kounis syndrome induced by inactivated SARS-COV-2 vaccine (inactivated CoronaVac from China) in Turkey,^18^ which would raise concern due to the national vaccine roll-out plan in China with reported delivery of more than 3.34 billion doses of SARS-COV-2 vaccine (inactivated virus vaccines) by the time of writing this report.^19^

These facts pose a higher risk of iatrogenic medication-related allergic reactions for Chinese patients. Therefore, the discrepancy between the existence of risk factors and very low incidence reports in China is likely to be explained, at least in part, by a missed, unrecognised and/or undiagnosed condition. Take our case, for example. The diagnosis could easily have been missed if we hadn’t probed into the history of a bee sting (which happened three days before presentation to the ED) and neglected its association with cardiac symptomatology in someone with high cardiac risk factors. Also, due to the relatively late onset of cardiac-type chest pain (34 hours after the sting) and late presentation for medical attention (36 hours after onset of chest pain), the patient was given a provisional diagnosis of aortic dissection in the local hospital.

In our ED the initial diagnosis was allergic myocarditis after a negative CT aortogram, due to the unawareness of this condition. Luckily, medical management of ACS was not delayed, in concurrence with treatment of the allergic reaction. An elective PCI was organised when a diagnosis of Kounis syndrome was suspected after admission into the cardiology ward, which allows for clear delineation of the coronary anatomy and a potentially lifesaving stent placement.

Not surprisingly, given the low incidence, there is a lack of guidelines for the treatment of ACS in the form of Kounis syndrome. The common practice involves therapies to cover both myocardial reperfusion/revascularisation and allergic reaction.^20^ Corticosteroids and antihistamines are beneficial for the alleviation of inflammation and are helpful for the reversal of coronary vasospasm in the type I variant.^21^ In patients with type II variants, treatment should be directed by ACS treatment guidelines, in addition to steroids and antihistaminics.^22^ The management of the type III variant would be much more complicated, even needing stent extraction when necessary.^23^

## Conclusion

The main lesson of this case is that high suspicion of Kounis syndrome is needed when there is an allergic history with cardiac symptomatology. Only with increased awareness of this entity can more prompt initiation of treatment be achieved. Although in the majority of patients, the onset of clinical symptoms and signs are within one hour of trigger exposure,^5^ it could also present as a subacute or even chronic course. Measuring serum cardiac enzymes and cardiac-specific troponins is helpful in determining the level of cardiac damage associated with allergic insults. Echocardiography and coronary angiography are also indispensable in measuring cardiac wall abnormalities and management of coronary stenosis. Perhaps Kounis syndrome has been overlooked and underdiagnosed in China and being more vigilant about this condition in clinical practice may not only improve patient care but also lead to the discovery of more causative factors in the future.
